# Antimicrobial and Antibiofilm Activity of UP-5, an Ultrashort Antimicrobial Peptide Designed Using Only Arginine and Biphenylalanine

**DOI:** 10.3390/ph11010003

**Published:** 2018-01-02

**Authors:** Ammar Almaaytah, Mohammed T. Qaoud, Gubran Khalil Mohammed, Ahmad Abualhaijaa, Daniel Knappe, Ralf Hoffmann, Qosay Al-Balas

**Affiliations:** 1 Department of Pharmaceutical Technology, Faculty of Pharmacy, Jordan University of Science and Technology, Irbid 22110, Jordan; mqaud@daad-alumni.de (M.T.Q.); gubrankhalil@daad-alumni.de (G.K.M.); 2 Department of Applied Biological Sciences, Faculty of Science and Arts, Jordan University of Science and Technology, Irbid 22110 Jordan; ahkhaled18@yahoo.com; 3 Institute of Bioanalytical Chemistry, Faculty of Chemistry and Mineralogy and Center for Biotechnology and Biomedicine, Universität Leipzig, Deutscher Platz 5, 04103 Leipzig, Germany; daniel.knappe@bbz.uni-leipzig.de (D.K.); ralf.hoffmann@bbz.uni-leipzig.de (R.H.); 4 Department of Medicinal Chemistry, Faculty of Pharmacy, Jordan University of Science and Technology, Irbid 22110, Jordan; qabalas@just.edu.jo

**Keywords:** antimicrobial peptide, biofilms, ultrashort peptide, resistant bacteria

## Abstract

The recent upsurge of multidrug resistant bacteria (MDRB) among global communities has become one of the most serious challenges facing health professionals and the human population worldwide. Cationic ultrashort antimicrobial peptides (USAMPs) are a promising group of molecules that meet the required criteria of novel antimicrobial drug development. UP-5, a novel penta-peptide, displayed significant antimicrobial activities against various standard and clinical isolates of MDRB. UP-5 displayed MICs values within the range of (10–15 μM) and (55–65 μM) against Gram-positive and Gram-negative bacteria, respectively. Furthermore, UP-5 displayed antibiofilm activity with minimum biofilm eradication concentration (MBEC) value as equal to twofold higher than MIC value. At the same inhibitory concentrations, UP-5 exhibited very low or negligible toxicity toward human erythrocytes and mammalian cells. Combining UP-5 with conventional antibiotics led to a synergistic or additive mode of action that resulted in the reduction of the MIC values for some of the antibiotics by 99.7% along a significant drop in MIC values of the peptide. The stability profile of UP-5 was evaluated in full mouse plasma and serum with results indicating a more stable pattern in plasma. The present study indicates that USAMPs are promising antimicrobial agents that can avoid the negative characteristics of conventional antimicrobial peptides. Additionally, USAMPs exhibit good to moderate activity against MDRB, negligible toxicity, and synergistic outcomes in combination with conventional antimicrobial agents.

## 1. Introduction

The uncontrolled, outspread and extended use of antibiotics in recent decades has led to an unbounded resistance development by bacteria against most conventional antibiotics. Antimicrobial resistance develops when microorganisms (such as bacteria, fungi, viruses, and parasites) start to combat the effectiveness of antimicrobial agents. As a result, these agents gradually become ineffective. The multidrug resistance (MDR) phenomenon has become one of the most serious challenges in the clinical management of human infections [1]. 

From an economic point of view, the patients with resistant infections increase the cost of health care due to the longer duration of illness and hospitalization. The requirement for additional tests and the use of more expensive drugs contributes further in increasing the cost of health care for those patients than patients with non-resistant infections [2]. This issue has turned into a main concern worldwide, as there is no clear medication strategy to handle these types of serious infections. Therefore, massive efforts have been applied in order to develop new antimicrobial agents with unique modes of actions and low potential of bacterial resistance development to manage multidrug-resistant related infections and the post-antibiotic era.

All species of living organisms produce antimicrobial peptides (AMPs), which play a vital function within the organism by acting as fast weapons in the innate immune system against infecting pathogens, such as bacteria, fungi, and yeast [3,4,5,6]. These peptides are usually less than 50 amino acid residues long, display net positive charges, and exhibit an amphipathic nature [7].

The mode of action of AMPs is significantly different when compared to conventional antibiotics that act by targeting specific sites within the microbial cell, such as enzymes, ribosomes, or bacterial DNA. On the other hand, the mode of action of AMPs is related to causing irreversible damage to the cell membrane, thus allowing the AMPs to permeate the cell membrane physically and rapidly. Due to this unique mechanism of action, the probability of bacterial cells developing microbial resistance is very low compared to the available conventional antibiotics [8]. Furthermore, many models were proposed in order to explain AMPs’ modes of action, these models are matched by the diversity of AMPs in sequences, structures, and lengths [4,9,10,11].

In general, the antimicrobial activity of AMPs is governed by two substantial merits: positive net charge and hydrophobic residues. Based on the previous structure-activity related studies [12,13,14], the cationic and hydrophobic residues should be in a properly balanced state in order to avoid eukaryotic toxicity. There is a strong belief that the initial binding of cationic AMPs with the lipopolysaccharide (LPS) layer or lipoteichoic acids that hold a negative charge in Gram-negative and Gram-positive bacteria, respectively, is an essential step. It is the first substantial step to govern the antimicrobial activity of cationic AMPs. After that, AMPs navigate into and permeate the inner phospholipid membrane. Furthermore, it is suggested that DNA is another potential target for AMPs where binding to DNA leads to inhibition of DNA replication and transcription without damaging the cell membrane of bacteria [15,16,17,18]. It is believed that the bacterial membrane is broken down through the “carpet” mechanism which results in piling up of these peptides on the microbial surface till reaching a sensitive threshold concentration that eventually leads to the devastation of the bacterial membrane in a detergent-like a manner [19,20]. 

Regrettably, several factors limit the use of native peptides as therapeutics, including mammalian cell toxicity which is considered as a major disadvantage for AMPs, very poor bioavailability due to poor absorption and low metabolic stability towards proteolytic enzymes in the gastrointestinal tract after oral ingestion, in addition to the rapid excretion through the kidneys and liver [21]. Furthermore, complex structure and relatively large size are important factors that limit the chemical synthesis of the natural antimicrobial peptides, so accordingly significant efforts and high costs are needed to synthesize them. The defensins group is an example of AMPs of such criteria that consists of 18–45 amino acids including six disulphide linked cysteines [3,22].

Despite of all these drawbacks, development of new therapeutics based on proteins and peptides has attracted interests and therefore efforts are oriented toward the development of peptidomimetics, a new concept that have shown great promise both in medicinal and organic chemistry. This approach is based on enhancing the bioavailability and activity of native peptides and proteins, which are considered as tools to develop drug candidates and new therapeutic classes, by introducing both functional and structural specific modifications while maintaining main features responsible for biological activity [21]. Apart from displaying greater oral bioavailability and biological activity, peptidomimetics may also show high cell selectivity than native peptides [23,24]. Peptidomimetics are divided into three classes depending on their functional and structural characteristics and these include [25]:

- Class I (structural mimetics): show a structural analogy with the native substrate in a well- defined spatial orientation, they carry all the functionalities responsible for the interaction with a receptor or an enzyme.
- Class II (functional mimetics): the analogy of this type is based on the interaction with the target enzyme or receptor regardless of their apparent structural similarity with the native compound.
- Class III (functional-structural mimetics): all the functional groups required for biological activity are mounted in a scaffoldwith a structure different from that of the starting compound.

Thyrotropin-releasing hormone (TRH) mimetic represents an elegant example of a peptidomimetic scaffold. This mimetic is based on a cyclohexane scaffold that replaces the peptide backbone. The pharmacophore represented by three functional groups are placed on the scaffold with maintaining spatial orientation of the amino acid side chains found in TRH hormone [26].

Current efforts for developing AMPs and enhancing their development towards the clinic are being focused on the design of a new sub-family called ultrashort antimicrobial peptides (USAMPs) which consist of three to eight amino acids. Several environmental and economic advantages are associated with the development of these new promising peptides. Chemically, they can be synthesized easily and purified simply. The amount of solvent and time required for synthesis is limited [27,28,29,30,31,32,33,34,35,36,37,38].

It was observed that according to previous studies aimed to design ultrashort peptides that using only two lysine or two ornithine amino acids to represent charged residues has led to loss of activity [28]. Further investigations showed that the antimicrobial and antibiofilm activity was significantly reduced as a result of replacing arginine with lysine residues due to favoring particular binding characteristics to bacterial membrane and DNA [39,40]. These results prove that membrane disruption is cation residue specific and furthermore cationic charges must arise from arginine and not lysine or ornithine, especially when the peptide is ultrashort in length. On the other hand, the hydrophobic moieties were represented by tryptophan which has a significant preference for the membrane interface compared to other hydrophobic amino acids. Also, biphenylalanine was used to represent hydrophobic moieties in order to enhance membrane insertion properties and using it led to reinforce peptide activity against methicillin-resistant *staphylococcus aureus* (MRSA) [28,41]. Furthermore, incorporation of non-natural amino acids was used as a main strategy to enhance peptide stability [42,43]. The biphenylalanine motif was reported as the shortest antimicrobial peptide-based agent. A severe damage to bacterial morphology and bacterial cell death were observed as a result of biphenylalanine interaction with bacterial cell membrane [44]. Besides that, the substitution of H by phenyl group in biphenylalanine is expected to incorporate a superior biological protection against enzymatic degradation compared to other non-natural motifs used alkyl substitution. This stability enhancement is secured via increasing basicity and decreasing polarity in addition to eliminate the predominance of *trans* versus *cis* peptide bond confirmation and some inter- and intramolecular hydrogen bonds [42,45]. 

Taking this into consideration, biphenylalanine (an unnatural amino acid) and arginine were used rationally in the present study to design a novel penta-peptide named UP-5. The peptide displayed potent antimicrobial activities against various standard and clinical isolates of MDRB. Additionally, UP-5 displayed potent antibiofilm activity with MBEC values as equal to MIC values. At the same inhibitory concentrations, UP-5 exhibited very low or negligible toxicity toward human erythrocytes and mammalian cells (Vero cells).

## 2. Materials and Methods

### 2.1. Peptide Design, Synthesis and Purification

The pentapeptide UP-5 (NH_2_-RBRBR-COOH, where B represents biphenylalanine and R represents arginine), was synthesized by G.L. Biochem (Shanghai, China), employing solid-phase methods and Fmoc chemistry. The purity and the identity (>98%) of the synthetic peptide were confirmed by reversed-phase high performance liquid chromatography (RP-HPLC) and electrospray Ionization (ESI-MS) mass spectrometry, respectively (supplementary material).

### 2.2. Antibiotics

Conventional antibiotics levofloxacin, chloramphenicol, rifampicin, and ampicillin were obtained from Sigma-Aldrich (Shanghai, China) and erythromycin from Sigma-Aldrich (St. Louis, MO, USA). According to the manufacture’s recommendations, the antibiotic powders and stock solution preparations were stored at the optimum temperature for each antibiotic.

### 2.3. Bacterial Strains

All bacterial susceptibility, biofilm, and synergistic assays of peptides, antibiotics, and peptides-antibiotics combinations used bacterial strains obtained from the American Type Tissue Culture Collection (ATCC, Manassas, VA, USA) that states the mechanism of resistance for each strain according to ATCC number. Gram-positive bacteria were represented by five strains, the wild type strain *Staphylococcus aureus* (ATCC 29213) and methicillin-resistant *S. aureus* (MRSA) (ATCC 33591) in addition to clinically isolated strains *S. aureus* (ATCC BAA-41& 43300) and *Enterococcus faecalis* (ATCC BAA-2316). The Gram-negative bacteria strains were represented by wild type strains of *Escherichia coli* (ATCC 25922 & 35218) and *Pseudomonas aeruginosa* (ATCC 27853) as well as the clinically isolated multidrug-resistant strain *P. aeruginosa* (ATCC BAA-2114). The bacteria were cultured onto Muller Hinton Agar (Scharlab, S.L, Barcelona, Spain). Mueller Hinton Broth (Oxoid Ltd., Basingstoke, UK) was used to prepare all bacterial suspensions and to dissolve the peptides and the antibiotics. The assay was performed in sterile 96-well polypropylene microtiter plates.

### 2.4. Bacterial Susceptibility Assays

All bacterial susceptibility assays were conducted according to the microbroth dilution method outlined by the Clinical and Laboratory Standards Institute (CLSI) guidelines [46,47].

#### 2.4.1. MIC and MBC Values of UP-5

Briefly, pre-sterilized MHB was used as a growth medium for organisms obtained from stock media with glycerol. Bacterial cells were grown overnight in MHB and diluted to 10^6^ CFU/mL in the same medium prior to use. For peptide, serial concentrations in the range of 0.5–100 μM were prepared. Then, 50 μL of each peptide concentration and 50 µL of diluted bacterial suspension were added to each well of a 96-well microtiter plate. Peptide solutions were prepared by dissolving the peptide in sterile MHB. Then, the plates were incubated overnight in a humidified atmosphere, and at 37 °C. Bacterial growth was determined by measuring the optical density (OD) at *λ* = 600 nm using an ELISA plate reader (Epoch, BioTek, Winooski, VT, USA) and the minimum inhibitory concentration (MIC) was determined. For the minimum bactericidal concentration (MBC), 10 μL was withdrawn from wells having various concentrations of the peptide, clear negative wells, and turbid positive control wells, and were allowed to grow after sub-culturing on pre-sterilized labeled agar plated for 24 h at 37 °C. The lowest concentration that led to having <0.1% viable cells (killing 99.9%) of the subcultures refers to the MBC value.

#### 2.4.2. MICs and MBC Determination of Individual Antibiotics

As described in the previous section, the MIC and MBC values of antibiotics employed in this study were determined against subjected bacterial strains via preparing different concentrations of each antibiotic. 

#### 2.4.3. MIC and MBC Determination for Peptide and Antibiotics in Combinations

According to the broth microdilution checkerboard technique [48], the MIC and MBC values of peptide-antibiotics combinations against wild type and multidrug resistant bacterial strains were determined. Serial concentrations of peptide and each antibiotic were prepared in the range of 0.005–100 μM. In each well of a 96-well microtiter plate, 25 μL of each peptide concentration were added to 25 μL of each antibiotic concentration which is followed by adding 50 μL of diluted bacterial suspension (10^6^ CFU/mL) to each well. The prepared concentrations of peptide and each antibiotic were arranged into rows and columns, respectively, in a 96-well microtiter plate according the checkerboard technique. Then as described previously (see Section 2.4.1), the procedure was followed. All MIC and MBC determinations of all assays were made in triplicate.

#### 2.4.4. FIC Determination

The fractional inhibitory concentration (FIC) is defined as the inhibitory concentration of the antimicrobial combination divided by that of the single antimicrobial component. To calculate FIC index for peptide-antibiotic combinations, the following equation was used:

(1) $$\mathrm{FIC}\;\mathrm{index}=\frac{\mathrm{MIC}\;\mathrm{of}\;\mathrm{drug}\;X\;\mathrm{in}\;\mathrm{combination}}{\mathrm{MIC}\;\mathrm{of}\;\mathrm{drug}\;X\;\mathrm{alone}}+\frac{\mathrm{MIC}\;\mathrm{of}\;\mathrm{drug}\;Y\;\mathrm{in}\;\mathrm{combination}}{\mathrm{MIC}\;\mathrm{of}\;\mathrm{drug}\;Y\;\mathrm{alone}}$$

The FIC indices were interpreted as follows: ≤0.5: synergistic activity, 0.5–1: additive activity, 1–4 indifferent, >4: antagonism. Interpretation and assessment of the FIC indices were performed according to the broth microdilution checkerboard technique [48].

### 2.5. Antibiofilm Assay

Biofilm formation relied on a published protocol employing the Calgary biofilm device (Innovotech, Edmonton, AB, Canada) [49,50]. Briefly, *S. aureus* (ATCC 33591) bacterial strain was incubated in MHB and cultures were diluted in the same medium to achieve a concentration of 10^5^ CFU/mL. Next, diluted bacterial suspension (150 µL) was added to the sterile 96 peg-lids on which biofilm cells can build up. Negative control lanes were prepared by adding 150 µL MHB to six wells. And then the pegs were incubated for 14–16 h under rotation of 125 rpm at 37 °C to allow biofilm formation on the purpose designed pegs. The pegs were rinsed twice with phosphate-buffered saline (PBS) to remove planktonic cells as a washing step. Each peg-lid was then transferred into a “challenge 96-well microtiter plate” containing 200 μL of different peptides concentrations and the peg-lids containing the biofilms were incubated for four hours at 37 °C under rotation at 125 rpm. After biofilm treatment with the challenge plate, the biofilms were re-washed twice with PBS then transferred into the recovery plate for eight hours. The minimum biofilm eradication concentration MBEC value is defined as the minimum concentration needed to inhibit the re-growth of biofilms after four hours of peptide treatment, washing twice with PBS and incubating in pre-sterilized MHB (recovery plate) for 8 h, using an ELISA plate reader λ = 600 nm. Additionally, the biofilms were assessed for their minimum bactericidal concentration (MBECb), this assay was conducted similar to MBC assay (see Section 2.4.1). MBECb parameter is defined as the lowest concentration able to eradicate 3log^10^ of the viable microorganisms in a biofilm (99.9% killing) after 8 h of incubation in recovery plates using the colony count method.

### 2.6. MTT Cell Proliferation Assay

Vero cells were seeded at 5 × 10^3^ cells per well in flat-bottomed 96-well plates, and the plate was incubated for 18–24 h at 37 °C under 5% CO_2_ to allow cell attachment on the bottom of the plates. Next day, different concentrations of the peptide were prepared as a gradient using growth medium as the diluent (150, 200, 250, 300, 400, 500, and 600 µM). The plates were incubated for 24 h at 37 °C under 5% CO_2_. Next, 30 μL (2.5 g/L) MTT solutions were added to all wells and the plates were incubated for 4–6 h. After incubation, the MTT-Peptide solution was removed and a 100 μL of DMSO was added to each well and mixed thoroughly by pipetting to dissolve the Formazan crystals at the bottom of the wells until a clear purple color was achieved. The plates were then placed on an ELx808™ Absorbance Microplate Reader (BioTek, Winooski, VT, USA) and the absorbance was measured at λ = 540 nm.

### 2.7. Erythrocyte Hemolytic Assay

Two milliliters of human blood were placed into a 50 mL centrifuge tube and centrifuged (3000× *g*). The supernatant was discarded, the cell pellet re-suspended in 48 mL of PBS, and centrifuged as above; this step was repeated twice. Finally, 2 mL of cell pellet were re-suspended in a sterile tube containing 48 mL PBS to reach a final concentration of 4% RBC. Different concentrations of the peptide were prepared (40, 60, 80, 100, 125, 150, 175, and 200 μM), and then 1 mL of each peptide concentration was added to 1 mL of erythrocyte suspension. Positive controls were prepared by adding 5 µL of 0.1% (Triton 100X) to 2 mL of 4% RBC suspension and negative controls were prepared by adding 2 mL of 4% RBC suspension only. All preparations were incubated for 60 min at 37 °C. Next, the tubes were gently vortexed and 1 mL of each sample were removed and placed into sterilized eppendorf tubes and then centrifuged (3000× *g*). Then, 200 µL of each supernatant was placed into a 96 well-plate. Absorbance was measured at λ = 450 nm with the aid of an ELISA reader [51]. The percentage hemolysis was calculated according to the following equation:

(2) $$\%\;\mathrm{Hemolysis}=\frac{A-\mathrm{AO}}{\mathrm{AX}-\mathrm{AO}}\times 100$$

where A is the optical density at 450 nm (OD_450_) of the peptide solution, AO is the OD_450_ of the negative control (0.9% NaCl) and AX is the OD_450_ of the positive control (0.1% Triton 10X).

### 2.8. Stability Assays

The stability assay of UP-5 relied on a previously reported stability test procedure [52] employing mouse plasma or mouse serum (PAA Laboratories, Pasching, Austria). Briefly, a stock solution of the peptide was prepared in 5% aqueous DMSO. The highest DMSO concentration was less than 0.5% after dilution in order to have a final concentration of peptide equal to 1 g/L. Each peptide was dissolved in plasma or serum at a final concentration corresponding to 75 µg/mL and incubated at 37 °C (750 rpm, Eppendorf, Hamburg, Germany). After different time periods, aliquots (47.5 µL) were taken and proteins were precipitated by addition of 150 µL of a mixture composed acetonitrile, water, and formic acid (89:10:1 by volume). The samples were incubated on ice for 45 min and centrifuged for 10 min at 13,000× *g* (Eppendorf, Hamburg, Germany). After transfer to a new reaction tube, the supernatant (170 µL) showed partial precipitation and was therefore centrifuged a second time. The second supernatant (155 µL) was collected and dried under vacuum. The samples were dissolved in 3% aqueous acetonitrile containing, 0.1% formic acid, and were analyzed using an Agilent 1100 HPLC system (Agilent Technologies, Santa Clara, CA, USA), coupled online to an Esquire HCT mass spectrometer (Bruker Daltonik GmbH, Bremen, Germany) equipped with an analytical Jupiter C_18_-reversed phase column (2 mm inner diameter, 150 mm length, 5 µm particle size, 125 nm pore size). Eluent A contained 0.1% formic acid in water and Eluent B was 60% aqueous acetonitrile containing 0.1% formic acid.

### 2.9. Statistical Analysis

All statistical analyses were performed using the GraphPad Prism 7 software (La Jolla, CA, USA) program and Microsoft Office excel 2016.

## 3. Results

### 3.1. Design of UP-5

**Figure 1:**
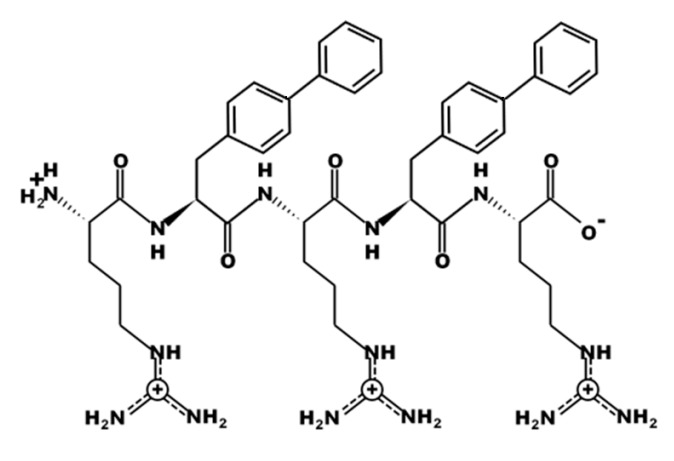
Chemical structure of UP-5.

UP-5 was designed rationally with the balance between the hydrophobic and charged moieties taken into account. Arginine (R) was chosen to represent the charged moieties, while biphenylalanine (B) was incorporated in between to represent the hydrophobic residues. Five amino acids residues represents the overall sequence of UP-5 (Mw = 932.15 g/mol) which exhibited a charge of +3. The structure of UP-5 is displayed in Figure 1.

### 3.2. In Vitro Antimicrobial Activity of UP-5

The in vitro antimicrobial activity of UP-5 was assessed against representative wild type and multidrug resistant bacterial strains of Gram positive and Gram negative bacteria (Table 1). UP-5 managed to inhibit the growth of wild type and methicillin resistant G+ bacterial strains of *S. aureus* (ATCC 29213 & 33591, respectively) with a MIC value of 10 μM. The other G+ bacterial strains including the two clinically isolated methicillin resistant strains *S. aureus* (ATCC 43300 & BAA-41) were less susceptible with MIC values of 15 μM. The vancomycin resistant bacterial strain *E. faecalis* (ATCC BAA-2316) was inhibited at a significantly higher MIC value of 70 μM. UP-5 was less potent against G− bacteria, *E. coli*, *P. aeruginosa*, with MIC values ranging from 55 to 65 μM.

Additionally, the bactericidal activity of UP-5 against all the subjected bacteria strains was evaluated by quantifying the minimum bactericidal concentration (MBC) (Table 1). All measured MBC values of UP-5 were equal to MIC values of the tested G+ and G− bacterial strains indicating that UP-5 is a bactericidal peptide. 

### 3.3. Antimicrobial Synergistic Assays

The antimicrobial activity of UP-5 in combination with five conventional antibiotics (levofloxacin, chloramphenicol, rifampicin, ampicillin, and erythromycin) was assessed against *S. aureus* (ATCC 29213), *S. aureus* (ATCC 33591), *S. aureus* (ATCC 43300), *S. aureus* (ATCC BAA-41), and *P. aeruginosa* (ATCC BAA-2114).

#### 3.3.1. Determination of the MIC and MBC of the Individual Antibiotics

The five studied antibiotics were tested against wild type and multidrug-resistant bacterial strains in order to determine the antimicrobial activity of each antibiotic against the employed bacterial strains. In addition to the MIC values, the bactericidal activity of the applied antibiotics was evaluated by determining the minimum bactericidal concentrations (MBCs). All reported data of MIC and MBC values of antibiotics are summarized in (Table 2). As reported, the MIC values of levofloxacin, rifampicin and ampicillin were on a par with the MBC values. This is an indication of the bactericidal activity of these antibiotics. On the contrary, a bacteriostatic activity was reported for chloramphenicol and erythromycin due to having higher MBC values when compared to their MIC values.

#### 3.3.2. Checkerboard Assay Results

As shown in (Table 2), the MIC values of peptide decreased dramatically, while calculating the percentage of dropping of MIC values of antibiotics in combination with UP-5 compared to MIC alone displayed a significant reduction (*p* value *p* ≤ 0.05). A reduction equal to 99.5% to rifampicin MIC value compared to MIC alone was reported when tested against the MDR strain *P. aeruginosa* (ATCC 2114), displaying the most synergistic behavior (FIC value = 0.46). Additionally, this combination led to an 87.5% reduction of rifampicin MIC value when tested against the *S. aureus* (ATCC 43300 and 33591) strains and displayed synergistic effects with FIC values equal to 0.45 and 0.13, respectively. On the other hand, according to FIC values, this combination led to indifferent effects when tested against the clinical isolate of *S. aureus* (ATCC BAA-41) strain, despite showing a 60% reduction of antibiotic MIC value compared to MIC alone, and *S. aureus* (ATCC 29213). The chloramphenicol-UP-5 combination led to a 93.7% and 70% reduction of antibiotic MIC values compared to the MIC alone when tested against *P. aeruginosa* (ATCC 2114) and *S. aureus* (ATCC BAA-41), respectively. Based on FIC values, this combination displayed only one synergistic effect against *P. aeruginosa* (ATCC 2114) with FIC value equal to 0.24, otherwise, it displayed additive effects. Combining levofloxacin with UP-5 against *S. aureus* (ATCC 29213 & 33591) strains led to a percentage of reduction of levofloxacin MIC value equal to 87.5% and 90%, respectively, compared to MIC alone. Levofloxacine-UP-5 combination exhibited synergistic effects against *S. aureus* (ATCC 29213, 43300 and 33591), additive effect against *P. aeruginosa* (ATCC 2114) and indifferent outcome when tested against *S. aureus* (ATCC BAA-41). A reduction of MIC value equal to 85.3% of ampicillin compared to MIC alone was obtained when tested against *S. aureus* (ATCC 33591) with FIC value equal to 0.27 that showed a synergistic effect. The ampicillin-UP-5 combination also displayed additive effects when tested against *P. aeruginosa* (ATCC 2114) and *S. aureus* (ATCC 43300) with FIC values equal to 0.86 and 0.62, respectively. The last combination (erythromycin-UP-5) displayed an additive effect with a 60% reduction of erythromycin MIC value compared to MI alone, FIC value was equal to 0.9. Other combinations displayed a reduction of 50% or less of antibiotics’ MIC values when combined with UP-5 against subjected bacterial species. In summary, out of the overall twenty-five antimicrobial combinations of UP-5 and the antibiotics, 32% were synergistic and 44% were additive against the tested bacterial strains while the other combinations did not show any antagonism effect according to the FIC index.

#### 3.3.3. Determination of MBCs of UP-5 and Antibiotics in Combinations

Determining the MIC values of UP-5 and antibiotics in combinations was followed by evaluating the bactericidal activity of those combinations. This issue was evaluated by measuring the minimum bactericidal concentration (MBC) against the subjected bacterial species included in this study. Both chloramphenicol and erythromycin, which exhibit bacteriostatic antimicrobial activity, displayed bactericidal activity in combination with UP-5. Thus, the MBC values of all antibiotics were equal to the MIC values in all combinations (see Table 2).

### 3.4. Antibiofilm Activity of UP-5

The antibiofilm activity of UP-5 against biofilm structures formed by the multidrug resistant strain *S. aureus* (ATCC 33591) was evaluated by two distinct techniques using the Calgary device: measuring the minimum biofilm eradication concentration (MBEC) and counting viable bacterial cell (MBCb) after treatment in accordance to the colony count method. As summarized in (Table 3), treatment of bacterial biofilm cells with peptide concentration equal 10 μM caused a reduction of viable biofilm cells to about 0.35% (99.65% killing) compared to negative control (untreated formed biofilm). The reported UP-5 concentration required to eradicate biofilm formation after 4 h of exposure at 37 °C was equal to 20 μM, a concentration that is twofold higher than the MIC value. This is a clear indication that UP-5 has a potent antibiofilm activity at relatively a low concentration. Additionally, the minimum UP-5 concentration needed to reduce the number of viable biofilm cells to almost zero (99.9% killing) (MBECb) against biofilm cells was also found equal to the MBEC value (20 μM) (Table 3). These results clearly display the antibiofilm potential of UP-5 and the innate antimicrobial resistance of biofilms towards ultrashort peptides. 

### 3.5. Hemolytic Assay

The hemolytic activity of UP-5 peptide was assessed against human erythrocytes. All the results are summarized in (Table 4). The obtained results indicate that only 1% hemolysis was reported after 1h of incubation with human erythrocytes at 100 μM concentration, which is equal to about a sevenfold higher concentration than UP-5 MICs (10–15 μM) against G+ and G− bacteria. Additionally, UP-5 didn’t cause a hemolytic effect over 4% even at 200 μM concentration. 

### 3.6. Cell Cytotoxicity

**Figure 2:**
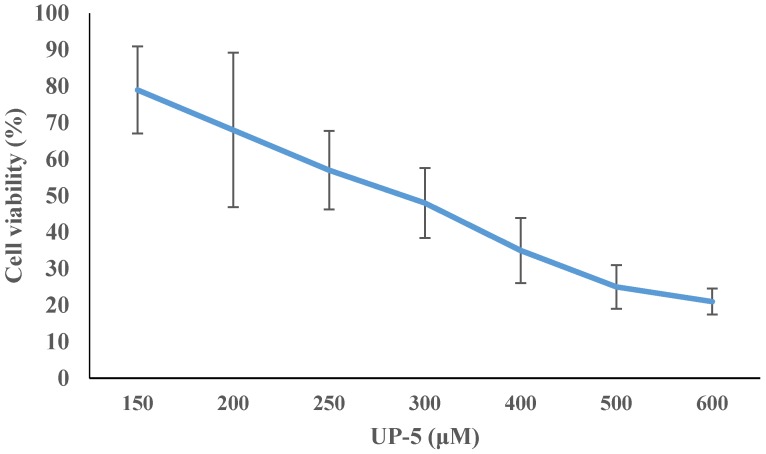
Cell viability (%) of Vero mammalian cells as a response to different UP-5 concentrations exposure. Bars represent the standard deviation (±SD). Values represent means of three different experiments.

The cytotoxicity and selectivity of UP-5 against eukaryotic mammalian cells was also assessed using MTT cell proliferation assay and employing Vero mammalian cells. Different peptide concentrations (150, 200, 250, 300, 400, 500 and 600 μM) were subjected against the employed cell line and the proliferation activity was studied. Cell viability (%) equal to 79% (killing 21%) of Vero cells was obtained at concentration of 150 μM concentration of peptide treatment. Reaching a concentration of 600 μM led to a killing of 79% of Vero cells. The relationship between cell viability (%) and the UP-5 concentrations was also determined (Figure 2), the IC_50%_ value of UP-5 against Vero mammalian cells was more than 250 μM, which is equal to about seventeen fold higher than UP-5 MICs (10–15) against G+ and G− bacteria. The results obtained from the hemolytic assay and cell toxicity assay indicate that UP-5 is exerting negligible toxicity against eukaryotic cells at the antimicrobial concentrations needed to inhibit bacterial growth. 

### 3.7. Stability Assay

**Figure 3:**
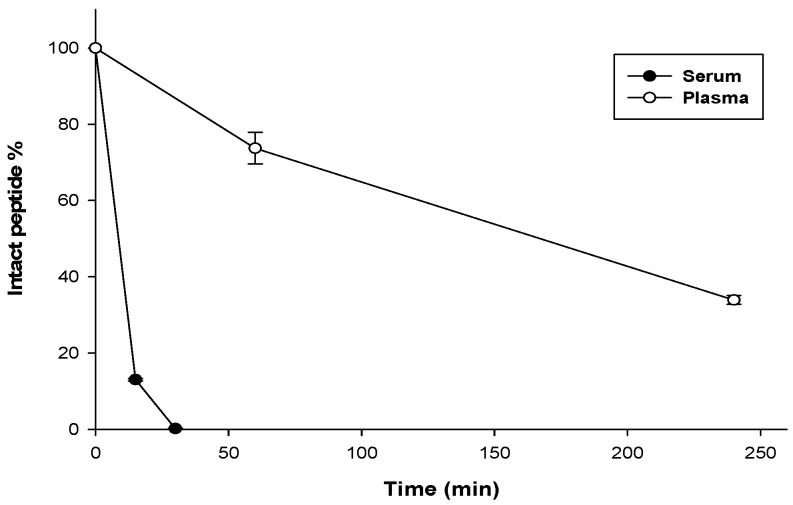
The serum and plasma stability profiles of UP-5. Samples were incubated at 37 °C with full mouse serum and plasma. Aliquots were taken at different time points. Results are the mean values ± SEM of three independent experiments.

The stability assay was used to provide preliminary information about the in vivo profiles of UP-5. To what extent the peptide has the ability to resist proteolytic degradation by blood enzymes was determined in the assay. As summarized in Figure 3, the stability of UP-5 in full mouse plasma was studied at different time points. The peptide amount was determined based on the peak areas relative to the initial peptide quantity at 0 min (set to 100%). It was reported that: after 1-h incubation in plasma, only 27% of initial amount was degraded (73% intact peptide) and at 4 h of incubation the peptide was degraded by 66% (34% intact peptide).

On the other hand, regarding the serum stability test, UP-5 was studied in full mouse serum at different time points (Figure 3). As reported, more than 80% of the initial peptide amount was degraded after 15 min and after 30 min, the peptide was totally degraded. These results indicate that UP-5 is more stable in plasma (t_1/2_ = 173 min = 2.9 h) than in serum (t_1/2_ < 15 min). This data indicates that the peptide suffers from high in vivo degradation and poor stability which would consequently limit the application of this peptide for topical antimicrobial therapy.

## 4. Discussion

‘‘Fight the resistance”, has become one of the most famous slogans launched by all the human health-care communities worldwide in view of the alarming emergence of infectious diseases as well as the increase in bacterial multi-resistance. In order to manage this issue, new antimicrobial agents using new modes of action have been in development to meet this imminent challenge [53]. AMPs represent a promising class of antimicrobial agents providing a lot of advantages compared to conventional antibiotics including the lower likelihood of resistance development, accompanied with high potency against multi-drug resistant strains. In the literature, over 600 AMPs have been reported as potent agents against multi-drug resistant bacteria (MDRB) and biofilm forming strains [54]. The last research efforts were directed to the introduction of novel or modified amino acids displaying several features which have represented a promising approach to develop valuable and effective peptidomimetics. For instance: replacing proteinogenic amino acids with their corresponding D-variants resulted in enhanced in vivo biological activity, introduction of a stereocenter at the β-position using β-methylamino acids has been reported to result in a conformational restriction of bioactive peptides and substitution of proline with 5,5-dimethylthiazolidine-4-carboxylic acid (an unnatural amino acid) resulted in 39% greater agonist activity toward angiotensin II, a key peptide in blood pressure regulation [21]. The current efforts are aimed at developing AMPs with ultrashort sequences (ultrashort peptides) based on a selection of specific amino acids that exhibit specific and unique characteristics suitable for antimicrobial activity. Those ultrashort sequences represent new alternative antimicrobial agents designed to mitigate and avoid the negative shortcomings of the long and short peptides that are reflected by the occurrence of high mammalian toxicity as well as high cost issues. The ultrashort peptides are cost effective as they can be chemically synthesized via simpler and less complex procedures [55].

The antimicrobial activity of AMPs is mainly governed through targeting the bacterial cell membranes. Targeting is facilitated by the notable hydrophilic positively charged residues of AMPs and the microbial negatively charged surfaces leading to pore formation and hence cell membrane lysis and death. As UP-5 displays a net cationic charge of +3, which carries a sufficient positive charge in order to create sufficient electrostatic attraction and hence achieves complete interaction with the negative head groups of phospholipids in the bacterial cell membrane and cationic residues of the peptide [28].

The charged moieties of UP-5 were represented by arginine since the side chain can interact through the formation of hydrogen bonds and also through electrostatic interactions with the negatively charged surface of bacteria. The arginine’s guanidine moiety as well as arginine side chain play a vital role for anchoring cationic AMP to the membrane surface via formation of a complex with the phosphate groups of the phospholipid bilayer [28,56]. 

The hydrophobic moieties of UP-5 were represented by the unnatural amino acid biphenylalanine, as well as the C5-chain of arginine amino acid. Insertion of two lipophilic residues created a sufficient ‘grip’ to these residues for the peptide, thus allowing the peptide to disrupt the bacterial membrane integrity after membrane insertion. The existence of at least two lipophilic residues will ensure a maximal thinning of the membrane at a certain radius around the peptide followed by membrane disruption [28]. 

As demonstrated in the present study, the MIC values of Gram-negative bacteria were significantly higher than the MICs of the Gram-positive bacteria; these differences could be explained due to differences in their respective cell wall compositions. Gram-negative bacteria contain a thin peptidoglycan lipid bilayer (~7–8 nm) which is a three-dimensional rigid structure consisting of a linear polysaccharide chains cross-linked by short peptides. and is also surrounded by an additional outer membrane of a lipopolysaccharide (LPS) layer while Gram-positive bacteria possess a thick peptidoglycan lipid bilayer (~20–80 nm) but lacks an outer layer of LPS [20,57,58]. The MBC values reported for UP-5 against subjected bacterial species were equal to the MIC values, which indicate that the peptides are bactericidal in nature.

The antimicrobial activities of UP-5 in different combinations with antibiotics were also evaluated. The results of the targeted MIC assay of five conventional antibiotics and UP-5 differed according to the bacterial strain; furthermore, the MIC values of the same antibiotics were significantly different from the MICs of the ultrashort peptide on the same strains. These differences can be explained due to differences in the mechanism of action that is suggested for the ultrashort peptides and the known mechanisms of action of the antibiotics used in the study. The antibiotics used in our study have different mechanisms of action such as inhibiting protein synthesis, interfering with nucleic acid synthesis, and blocking cell wall synthesis while, the antimicrobial activity of AMPs is mainly governed by cell membrane disruption and destabilization that leads to increasing membrane permeability through pore formation, which ultimately causes cell lysis and death [59,60,61].

As many of the tested combinations displayed synergistic activities or at least additive effects, this behavior suggests that the possible membrane targeting of UP-5 might have potentiated the effects of antibiotics. However, the mechanisms of the synergistic effects of the peptide- antibiotic combinations are still unclear. One of the proposed mechanisms for the synergistic effect was the destruction, permeabilization, and pores formation effects of AMPs against bacterial membranes which facilitates and enhances the intracellular entry of antibiotics. Additionally, it is suggested that the peripheral membrane proteins essential for cell wall biosynthesis and respiration are mainly delocalized, cellular energy is limited and cell wall integrity is undermined by the small cationic antimicrobial peptides. All antibiotics used act intracellularly except ampicillin, which functions via inhibiting bacterial cell wall synthesis. Therefore, combining antimicrobial agents with different mechanisms of action usually leads to the enhancement of the performance of each combination’s constituent, and causes bacterial cell disruption rapidly and efficiently [62,63,64]. Based on these studies data, we propose that these proposed mechanisms are the most probable mechanisms for peptide-antibiotic combinations. 

In regards to the results of combining UP-5 with five different types of antibiotics, the MIC values for both antibiotics and peptides in combination decreased dramatically (Table 2) in most groups. It is reported in general that 10 to 1000-folds higher concentrations of conventional antibiotics are required to restrain biofilm growth effectively, when compared with planktonic cells due to the presence of the extracellular lipopolysaccharide matrix surrounding the biofilms [65]. The development of antibiofilm therapeutics has generally been based on enhancing the dispersion of biofilm pioneer cells, inhibition of adhesion and interfering with the quorum sensing system. In the present study, the antibiofilm activity was determined and UP-5 displayed potent antibiofilm activity Compared to previous rare studies that investigated the antibiofilm activities of different ultrashort peptides. For instance: the MBEC values of OOWW-NH2 and C6-OOWW-NH2, two ultrashort peptides previously designed by Bisht et al., were sometimes 128-fold higher than the MIC values [30,65]. This is a clear indication that UP-5 is a potent antibiofilm agent that displays significant antibiofilm activity at relatively low concentrations.

Based on previous studies, some AMPs such as melittin and newly synthesized antimicrobial peptides (long and short sequences) displayed hemolytic and cytotoxicity activity ranging from moderate to high rates toward eukaryotic cells [66,67,68] while, very low or negligible toxicity was reported for UP-5. The low toxicity of the ultra-short peptide is thought to be due to its low hydrophobicity and short size. Furthermore, the high cationic charges of UP-5 (+3 net charges) mainly participated in minimizing the toxicity towards eukaryotic cells. Thus, mass charge plays a major role in creating sufficient electrostatic attraction and hence targeting the negative head groups of the bacterial cell membrane and consequently increasing the peptide’s selectivity. This higher selectivity towards bacterial cell membrane is thought to be due to having a higher proportion of anionic phospholipids compared to the zwitterionic phospholipids in eukaryotic cells [12,69,70,71]. Additionally, it is suggested that erythrocytes are protected against the peptides lysis via neutralization of the cationic peptides with the negatively-charged sialic acid that is available abundantly and is located about 80 Å above the cells’ surface. 

The stability of UP-5 was studied in full mouse plasma and serum at different time points. The results indicated that UP-5 has a better stability profiles in plasma (t_1/2_ = 173 min = 2.9 h) than in serum (<15 min). The mechanism of this enhanced stability profiles in plasma than in serum is unclear but, it’s proposed that the presence of clotting factors and fibrinogens in blood plasma is playing a major rule for maintaining peptide intact and protecting cleavage sites. 

In a previous study in accordance to our results, Nguyen et al., investigated the stability profiles of short AMPs that are rich in arginine and tryptophan residues. It is reported that short peptide sequences usually display low stability profiles (h_1/2_ < 2 h) and they are considered susceptible agents for proteolytic enzymes especially that they exhibit a linear conformation [72]. In the present study, UP-5 displayed a better stability profile in plasma (h_1/2_ > 2 h) compared to the other short peptides reported in literature. This enhancement proves that incorporation of non-natural amino acids have a positive effect against enzymatic degradation. However, that degradation pattern is not yet optimal for systemic delivery and places restrictions on the peptide to be applied in topical antimicrobial applications only.

## 5. Conclusions

Herein, we report the design of a novel pentapeptide named UP-5, which displayed significant antibacterial activity against wild type and clinical isolates of multidrug resistant strains, including MRSA, with a bactericidal mode of action. Additionally, UP-5 displayed potent antibiofilm activity with negligible hemolytic activity and cytotoxicity towards human erythrocytes and mammalian Vero cells. The UP-5 combinations with conventional antibiotics led to a remarkable reduction of the peptide and antibiotics’ MIC values that usually were represented by a synergistic or additive outcome. The ultrashort peptide displayed better stability profiles in plasma than in serum. All these results indicate that UP-5 is a promising antimicrobial agent against both planktonic and biofilm forming MDR G+ and G− bacterial species and could have potential for developing topical antimicrobial applications.

## Figures and Tables

**Table 1:** Minimum inhibitory concentrations (MIC) and minimum bactericidal concentrations (MBC) of UP-5 against the subjected bacterial species.

Bacterial Species	ATCC #	MIC Value (μM)	MBC Value (μM)
*Staphylococcus aureus*	29231	10	10
*Staphylococcus aureus*	43300	15	15
*Staphylococcus aureus*	BAA-41	15	15
*Staphylococcus aureus*	33591	10	10
*Enterococcus faecalis*	BAA-2316	70	70
*Escherichia coli*	25922	55	55
*Escherichia coli*	35218	60	60
*Pseudomonas aeruginosa*	27853	65	65
*Pseudomonas aeruginosa*	BAA-2114	55	55

**Table 2:** Minimum inhibitory concentrations (MICs) and minimum bactericidal concentration (MBCs) of the individual antibiotics; MICs of UP-5 and the antibiotics in combination; Percentage reduction of antibiotic MIC values in combination with UP-5 compared to the individual MICs; the fractional Inhibitory concentrations (FIC) indices for the antimicrobial combinations against tested bacterial species.

Bacterial Species	Antibiotics				UP-5	FIC Index
		MIC/MBC Alone (μM)	MIC in Combination (μM)	Reduction in MIC (%)	MIC in Combination (μM)	
*S. aureus* ATCC 29213	Levofloxacin	0.5/0.5	0.05	90	0.125	0.11
	Chloramphenicol	20/30	10	<50	2.5	0.75
	Rifampicin	0.025/0.025	0.025	<50	10	2
	Ampicillin	2.5/2.5	2.5	<50	0.25	1.025
	Erythromycin	0.5/1.5	0.5	<50	10	2
*S. aureus* ATCC 33591	Levofloxacin	10/10	1.25	87.5	0.125	0.13
	Chloramphenicol	130/150	65	50	5	1
	Rifampicin	0.04/0.04	0.005	87.5	0.125	0.13
	Ampicillin	85/85	12.5	85.3	1.25	0.27
	Erythromycin	8/10	8	<50	0.125	1
*S. aureus* ATCC 43300	Levofloxacin	20/20	5	<50	0.025	0.25
	Chloramphenicol	15/20	7.5	<50	7.5	1
	Rifampicin	1/1	0.125	87.5	5	0.45
	Ampicillin	20/20	2.5	<50	7.5	0.62
	Erythromycin	100/150	100	<50	7.5	1.5
*S. aureus* ATCC BAA-41	Levofloxacin	10/10	10	<50	15	2
	Chloramphenicol	25/30	7.5	70	7.5	0.8
	Rifampicin	0.005/0.005	0.002	60	15	1.4
	Ampicillin	40/40	20	<50	7.5	1
	Erythromycin	350/400	125	64.3	15	1.35
*P. auroginosa* ATCC BAA-2114	Levofloxacin	12/12	5	58.3	20	0.78
	Chloramphenicol	200/325	12.5	93.7	10	0.24
	Rifampicin	50/50	0.25	99.5	25	0.46
	Ampicillin	>500/>500	250	>50	20	0.86
	Erythromycin	125/150	50	60	27.5	0.9

**Table 3:** The antibiofilm activity of UP-5 against a standard resistant *S. aureus* (ATCC 33591) strain.

Peptide Concentration (μM)	Viable Biofilm Cells (%)	±SD
100	0	0.0002
80	0	0.0005
60	0	0.0006
40	0	0.002
30	0.054	0.005
20	0.108	0.03
10	0.35	0.09
MBEC value	20	
MBCb	20	

**Table 4:** Hemolytic activity of UP-5 against human erythrocytes. The results were recorded at λ = 450 nm.

Hemolysis (%)	Concentration (μM)	±SD
0.09	40	0.0005
1.01	60	0.004
1.04	80	0.001
1.04	100	0.0005
2.06	125	0.002
3.0	150	0.001
3.04	175	0.001
3.09	200	0.01

## Supplementary Materials

The supplementary material incorporating HPLC analysis and Mass spectra pf UP-5 are available online at www.mdpi.com/1424-8247/11/1/3/s1, Figure S1: Analytical RP-HPLC chromatogram of the peptide UP-5, Figure S2: Positive electrospray ionization (ESI) mass spectrometric (MS) analysis of the peptide UP-5.
