# Supplementary material for: Antimicrobial and Antibiofilm Activity of UP-5, an Ultrashort Antimicrobial Peptide Designed Using Only Arginine and Biphenylalanine
Source: Pharmaceuticals (Basel). 2018 Jan 2;11(1):3. doi: 10.3390/ph11010003 (PMC5874699; doi:10.3390/ph11010003)

**Supplementary Material**

**Figure 1.** Analytical RP-HPLC chromatogram of the peptide RBRBR.


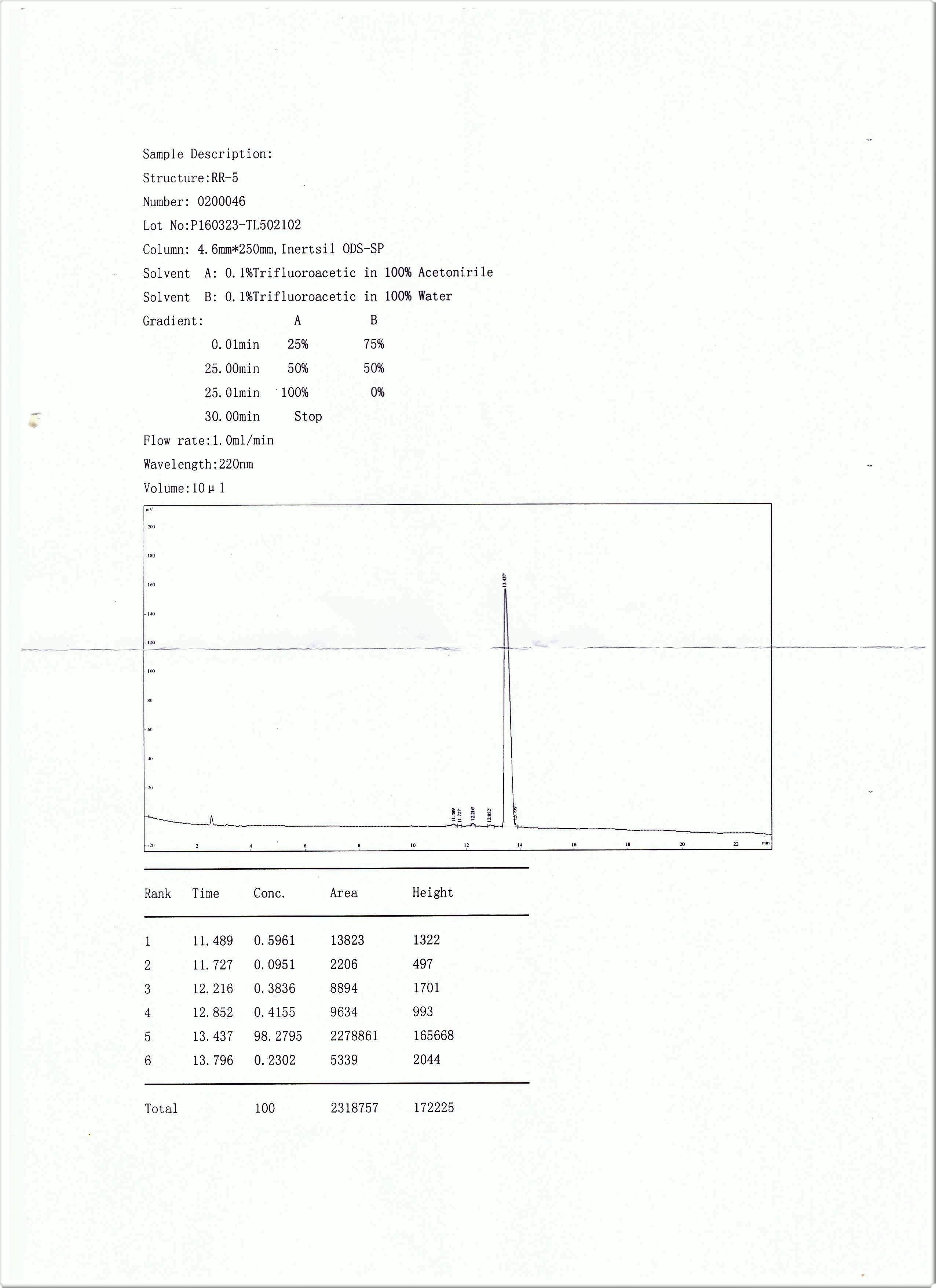


**Figure 2.** Positive electrospray ionization (ESI) mass spectrometric (MS) analysis of the peptide RBRBR showing major peaks in +1 charge state of 933.28 Da.


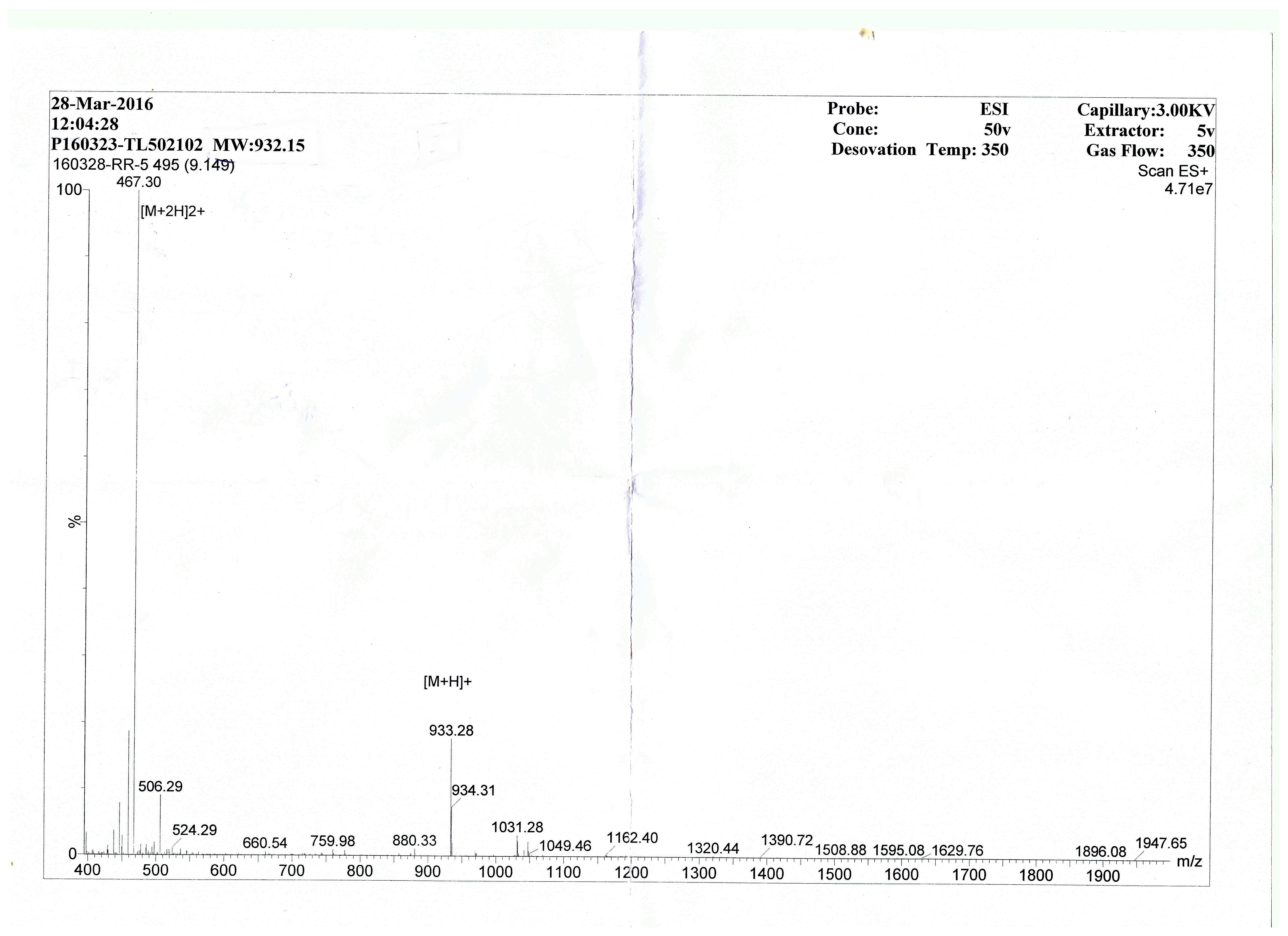

Supplement: Supplementary file 1 [file pharmaceuticals-11-00003-s001.doc]
